# Rhabdomyosarcoma of the Upper Lip in an Adult Patient

**DOI:** 10.1155/2015/508051

**Published:** 2015-04-21

**Authors:** Bengu Cobanoglu, Mustafa Simsek, Serkan Senol

**Affiliations:** ^1^Department of Pathology, Goztepe Training and Research Hospital, Istanbul Medeniyet University, 34722 Istanbul, Turkey; ^2^Department of Anesthesiology and Reanimation, Dr. Siyami Ersek Thoracic and Cardiovascular Surgery Training and Research Hospital, 34668 Istanbul, Turkey

## Abstract

Rhabdomyosarcoma (RMS) is a high-grade, malignant mesenchymal neoplasm. These tumors represent the most common soft tissue sarcomas of children and adolescents. RMS is uncommon on the lip and it is rarely seen in adults. Here, we report a rare case of primary RMS, embryonal type, occurring on the upper lip in a 27-year-old female.

## 1. Introduction 

Rhabdomyosarcoma (RMS), a malignant soft tissue neoplasm of skeletal muscle, was first described by Weber in 1854. It is the most common soft tissue sarcoma of childhood, accounting for 10% to 20% of all malignant solid tumors [1]. It also accounts for 4–8% of all malignant tumors in children under 15 years of age [2]. RMS occurs frequently in the head and neck areas, with the most frequently affected sites being the orbit, paranasal sinuses, soft tissues of the cheek, and the neck. Oral RMS is rare, and when it occurs, it is more frequently found in the soft palate [2–4].

We report a rare case of primary RMS, embryonal type, occurring on the upper lip in a 27-year-old female.

## 2. Case Report

A 27-year-old female was referred for evaluation of reddish in color, erythematous, nodular lesion on the upper lip. Physical examination of the patient revealed a painful mass, firm in consistency, measuring about 0.7 × 0.5 cm. No lesions were present on the mucosal surface and the mass did not cross the midline of face. The mass simulated a vascular lesion and was clinically diagnosed as a hemangioma. There was no lymphadenopathy. The remainder of the systemic examination was normal.

**Figure 1:**
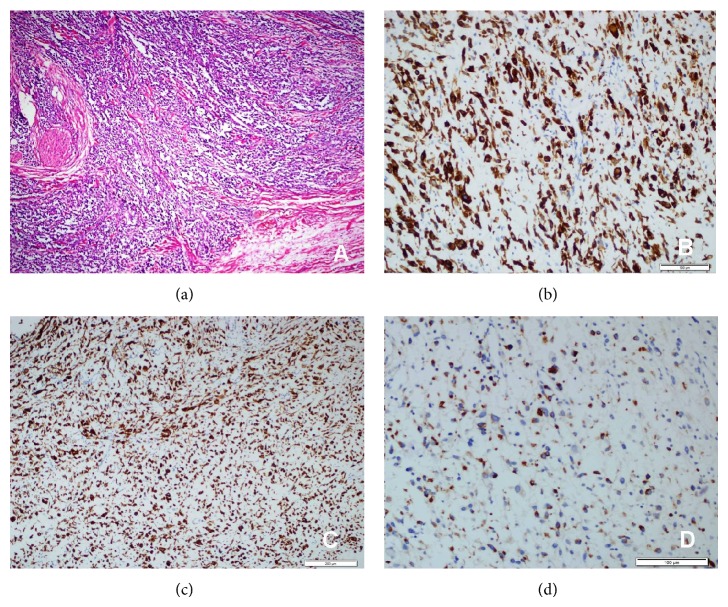
(a) Rhabdomyosarcoma (HEX100); (b) tumor cells are strong positive ones with myo-D1 (myo-D1X200); (c) there was also strong positivity with vimentin (vimentin X 100); (d) there was focal positivity for desmin (desmin X200).

An excisional biopsy was performed under local anesthesia. Material was excised and examined histopathologically. Light microscopy examination of hematoxylin and eosin stained sections showed small round cell tumor morphology. Tumor cells appeared round to oval, with hyperchromatic nuclei, scanty pale cytoplasm, and inconspicuous nucleoli (Figure 1(a)). The histopathologic features of the cells indicated a differential diagnosis comprising RMS, Ewing's sarcoma, malignant melanoma, or an epithelial tumor.

Immunohistochemical tests were performed with antibodies against vimentin, myogenin, myo-D1, desmin, muscle-specific actin, chromogranin, CD99, EMA, S 100, HMB 45, and Pan CK. In our case, among these markers, vimentin, myo-D1, muscle-specific actin, and myogenin revealed strong positive staining (Figures 1(b) and 1(c)), whereas no immunoreactivity to chromogranin, CD99, EMA, S 100, HMB 45, and Pan CK was detected. Focal positivity was evident for desmin (Figure 1(d)). Therefore, the tumor was diagnosed as embryonal RMS and verified by an immunohistochemistry panel. Surgical margins were free of tumor. She was started on adjuvant systemic chemotherapy. After 36 months of follow-up, she had no recurrences.

## 3. Discussion

RMS is a rare malignant lesion more common in children [5]. The incidence of RMS is highest in children aged 1–4 years, lower in children aged 10–14 years, and lowest in those aged 15–19 years. RMS is rare in adults, accounting for <1% of all malignancies [6, 7]. Our patient was 27 years of age; the occurrence of RMS at this age is extremely rare.

RMS occurs most often in the head and neck region, genitourinary tract, retroperitoneum, and extremities [8]. The orbit, nasal cavity, and nasopharynx account for about 30% of all head and neck RMS. The most common site of involvement in the oral cavity is the tongue, followed by the soft palate, hard palate, and buccal mucosa [9]. The lip is a relatively uncommon site for this tumor [10].

The head and neck RMSs are divided into two categories: parameningeal and nonparameningeal. The parameningeal type includes middle ear, paranasal sinus, nose, nasopharynx, mastoid, and pterygopalatine fossa RMSs, while the nonparameningeal type consists of scalp, orbit, oropharynx, oral cavity, larynx, and parotid gland RMSs [3]. Oral RMSs are classified within the nonparameningeal group of tumors and they have better prognosis [11]. Histologically, RMS belongs to the “small round cell tumor” group. Several different histological subtypes of RMS exist; each has different clinical and pathological characteristics.

The prognosis and clinical behavior of the tumor also partially depend on the histologic subtype. Multiple classification systems have been proposed for subclassifying these tumors. The most recent classification system, the “International Classification of Rhabdomyosarcoma,” was created by the Intergroup Rhabdomyosarcoma Study [12]. According to this system, four subtypes of RMS were established: (1) botryoid and spindle cell RMS; (2) embryonal RMS, generally having a superior prognosis; (3) alveolar (including the solid-alveolar variant) RMS, generally having a poor prognosis; and (4) undifferentiated sarcoma, also generally having a poor prognosis. RMS with rhabdoid-like features has been added to this classification, but this subtype and its prognosis are not clear [13]. Molecular pathology methods are applied for classification of RMS. Thus, criteria with greater objectivity for prognosis have been obtained based on genetic differences between the subtypes of RMS. For example, patients with the variant PAX7-FKHR translocation have a more favorable prognosis than do those with the more common PAX3-FKHR translocation [14].

RMS can be mistaken clinically for hemangioma, neuroblastoma, hematoma, and other sarcomas [10]. Our patient was initially diagnosed with hemangioma because the overlying mucosa was erythematous and shiny. In our case, the histologic type of RMS was diagnosed as embryonal according to histological and immunohistochemical findings. Among the immunohistochemical markers, vimentin, myo-D1, muscle-specific actin, and myogenin revealed strong positive staining. Myogenin is considered a sensitive and specific marker for RMS, as it is more specific than desmin and muscle-specific actin and more sensitive than myoglobin [15]. We could not conduct a molecular study for our case because this method was too expensive for the patient.

Early diagnosis is important. An early diagnosis of RMS and appropriate treatment can be highly effective. Treatment options include excision, chemotherapy, and radiotherapy. When the tumor can be totally removed, as in our patient, surgical excision is the most effective choice. If the tumor is huge and total excision is not possible, then a biopsy should be done for diagnosis. After diagnosis, chemotherapy is administered, thereby permitting localized control by a second surgery and/or radiotherapy [16].
